# A panel based on three-miRNAs as diagnostic biomarker for prostate cancer

**DOI:** 10.3389/fgene.2024.1371441

**Published:** 2024-05-16

**Authors:** Siwei Chen, Chong Lu, Shengjie Lin, Chen Sun, Zhenyu Wen, Zhenjian Ge, Wenkang Chen, Yingqi Li, Pengwu Zhang, Yutong Wu, Wuping Wang, Huimei Zhou, Xutai Li, Yongqing Lai, Hang Li

**Affiliations:** ^1^ Department of Urology, Peking University Shenzhen Hospital, Institute of Urology, Shenzhen Peking University-The Hong Kong University of Science and Technology Medical Center, Shenzhen, China; ^2^ Shenzhen University Health Science Center, Shenzhen, China; ^3^ The Fifth Clinical Medical College of Anhui Medical University, Hefei, China; ^4^ Shantou University Medical College, Shantou, China; ^5^ Peking University Health Science Center, Beijing, China

**Keywords:** prostate cancer, microRNA, target gene, biomarker, diagnosis

## Abstract

**Background:** Prostate cancer (PCa) is one of the most prevalent malignancies affecting the male life cycle. The incidence and mortality of prostate cancer are also increasing every year. Detection of MicroRNA expression in serum to diagnose prostate cancer and determine prognosis is a very promising non-invasive modality.

**Materials and method:** A total of 224 study participants were included in our study, including 112 prostate cancer patients and 112 healthy adults. The experiment consisted of three main phases, namely, the screening phase, the testing phase, and the validation phase. The expression levels of serum miRNAs in patients and healthy adults were detected using quantitative reverse transcription-polymerase chain reaction. Receiver operating characteristic (ROC) curves and the area under the curve (AUC) were used to evaluate the diagnostic ability, specificity, and sensitivity of the candidate miRNAs.

**Result:** Eventually, three miRNAs most relevant to prostate cancer diagnosis were selected, namely, miR-106b-5p, miR-129-1-3p and miR-381-3p. We used these three miRNAs to construct a diagnostic panel with very high diagnostic potential for prostate cancer, which had an AUC of 0.912 [95% confidence interval (CI): 0.858 to 0.950; *p* < 0.001; sensitivity = 91.67%; specificity = 79.76%]. In addition, the three target genes (DTNA, GJB1, and TRPC4) we searched for are also expected to be used for prostate cancer diagnosis and treatment in the future.

## Introduction

Prostate carcinoma (PCa) is one of the most common malignant tumors of the male genitourinary system, and its incidence and mortality rates rank second and fifth among male malignant tumors in the world, respectively. In 2022, about 268,490 individuals will be diagnosed with PCa in the United States, and about 34,500 will die of PCa (Sung et al., 2021). And in China, the status of PCa is not optimistic, showing a trend of increasing year by year. The main reasons for this are: the aging of the social population, changes in people’s lifestyles and the popularization of disease screening (Cao et al., 2021). Prostate carcinoma poses a considerable burden to middle-aged and older men, and its pathogenesis and etiology remain largely unexplained (Eeles et al., 2014). To date, DRE (digital rectal examination) and PSA (prostate-specific antigen) are still the most commonly used clinical methods for screening and assisting in the diagnosis of PCa. Still, due to the large number of interfering factors affecting the results of PSA and its low specificity and sensitivity, it often leads to overdiagnosis and overtreatment (Oesterling, 1991). Invasive prostate biopsy is the gold standard for diagnosing PCa, however, the positive rate of biopsy tissue is not high, and there is a risk of repeated puncture, and the detection rate of PCa is only 20% when the PSA level is between 4 and 10 ng/mL (Barcelo et al., 2019; Rodriguez Cabello et al., 2022). In summary, it is urgent to find a diagnostic biomarker that is non-invasive, specific and sensitive.

MicroRNAs (miRNA) are small, noncoding, single-stranded RNAs found in extracellular fluids such as plasma, serum, tears, and urine, which mediate post-transcriptional gene regulation by binding to and repressing specific mRNA targets (Weber et al., 2010). It is currently believed that miRNAs are involved in almost all cellular regulation and that up or downregulation of their expression is associated with the development of many human diseases (Krol et al., 2010). For example, it has been shown that miRNAs have a unique expression profile in the lungs and miRNA dysregulation is observed in most lung diseases, including lung cancer (Alipoor et al., 2016). The miR-199a has also been shown to have decreased expression in serum of patients with hepatocellular carcinoma, which can be considered as a potential biomarker (Wu et al., 2020). Two miRNAs (miRNA 206 and miRNA 574-5p) have also been shown to be overexpressed in coronary artery disease and have diagnostic value (Zhou et al., 2015). The miRNAs are approximately 90% circulating miRNAs, with the remaining 10% being present in exosomes, and studies have demonstrated that plasma miRNAs are stabilized by resistance to plasma RNase. Furthermore, the expression levels of similar miRNAs in the plasma and serum are not significantly different (Mitchell et al., 2008). In other words, serum miRNAs are very stable and easy to detect, and miRNAs may be aberrant in the early stages of the disease. Therefore, miRNA is considered a promising potential non-invasive biomarker for tumor diagnosis and prognosis.

This experiment gradually verifies the diagnostic ability, specificity and sensitivity of candidate miRNAs. After a screening phase, a testing phase and a validation phase, three miRNAs (miR-129- 1-3p, miR-381-3p and miR-106b-5p) with markedly aberrant expression in prostate cancer were selected from the initial ten candidate miRNAs. Compared with a single miRNA, we recently found that combining miR-129-1-3p, miR-381-3p and miR-106b-5p into a diagnostic panel has a high diagnostic ability, as well as solid specificity and high sensitivity, the AUC of three-miRNA panel was 0.912 (95% CI: 0.858 to 0.950; *p* < 0.001).

## Materials and methods

### Subjects and ethical review

One hundred and twelve patients who were diagnosed with prostate cancer after pathological gold standard diagnosis and had not received antitumor therapy from June 2017 to June 2021 in the Department of Urology of Shenzhen Hospital of Peking University were selected. 112 healthy adult men with no previous tumor-related medical history and complete clinical data confirmed by physical examination in our hospital during the same period were selected as the healthy control group. These are presented in Table 1. After enrollment, all participants collected 5–10 mL of circulating blood on an empty stomach in the morning and placed it in a sterile anticoagulation tube, and the serum obtained after centrifugation was stored in a refrigerator at −80°C for later use. The study was reviewed by the Ethics Committee of Peking University Shenzhen Hospital. Each participant has read and signed the informed consent form.

**TABLE 1:** Demographic manifestation of 224 participants (PC patients and HCs).

	Testing stage			Validation stage		
	(*n* = 56)			(*n* = 168)		
	PC	HCs		PC	HCs	
Total number age at diagnosis	28	28		84	84	
	67.6 ± 7.0	64.9 ± 9.5	*p* = 0.586	67.7 ± 9.5	66.0 ± 8.9	*p* = 0.243

In the two stages, statistical comparison by Wilcoxon-Mann Whitney test showed that there was no significant difference in age between PC, and HCs.

### Study method

**FIGURE 1:**
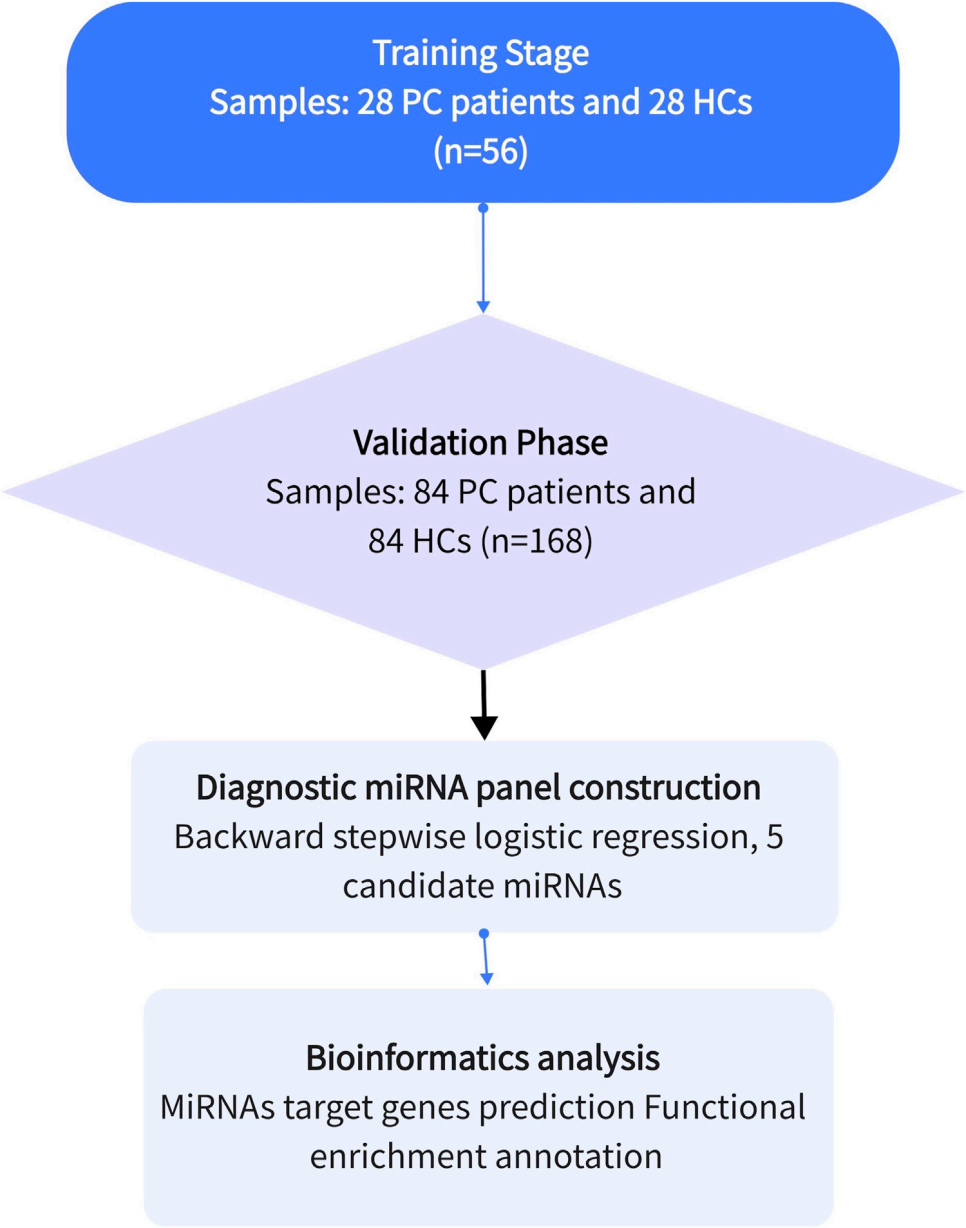
The general experimental procedure of this study. Determining the diagnostic value of candidate miRNAs through the training and validation phases. Apply the selected miRNAs to construct a diagnostic panel to improve the diagnostic capability, and finally perform bioinformatics analysis.

This study focused on identifying miRNAs with significantly abnormal expression levels in the tissues of prostate cancer patients through a three-stage case-control study. This is shown in Figure 1. Firstly, in the screening phase, we searched for differentially expressed miRNAs in the Gene Expression Omnibus and Pubmed databases, which are related to PCa. Then, in the testing stage, the serum specimens from 28 PCa patients and 28 healthy controls (HCs) were selected further to confirm the expression levels of candidate miRNAs by qRT-PCR. Filtering criteria were: *p*-value < 0.05 and fold change (FC) of >2 or < -2. In the validation phase, we used 168 serum samples (84 PCa and 84 HCs) to verify the results of the previous phase. The expression level and diagnostic ability of the candidate miRNAs were confirmed by bioinformation analysis and survival analysis, and the diagnostic panel were composed of miRNAs to improve the diagnostic sensitivity and specificity.

### RNA extraction and detection

Blood specimens were collected early in the morning from all participants while fasting, and the blood specimens were collected into sterile anticoagulant tubes spiked with 2 μL of miR-54, which controls the degree of variability during the extraction and purification phases. Total RNA was extracted with the RNA reagent TRIzol according to the manufacturer’s instructions, followed by diluting of the total RNA with 20 μL of RNase-free water and storage at −80°C pending subsequent use. We used a NanoDrop 2000 spectrophotometer to detect the concentration and then reverse transcribed the miRNA with specific primers from the Bulge-Loop miRNA qRT-PCR primer set to obtain the cDNA and used it as a template for a real-time polymerase chain reaction in a Light Cycler 480 Real-Time PCR System. The conditions under which the reaction occurs are set: 95°C for 15 s, followed by 60 s at 60°C, and finally at 70°C for 15 s. The reaction was repeated for 45 cycles.

### Bioinformatic analysis

MiRWalk 3.0 is a comprehensive miRNA target gene database that provides validated and predicted miRNA-regulated target genes. Our criteria for target gene selection are: he selected target genes can predict at least two or more candidate miRNAs. The selected target genes were then subjected to Gene Ontology functional annotation and Kyoto Encyclopedia of Genes and Genomes pathway enrichment analysis using the Enrichr database (http://amp.pharm.mssm.edu/Enrichr/).

### Statistical analysis

The data from this study were analyzed by SPSS software and were presented as mean ± standard deviation or as numbers and percentages. The ΔΔCT method was used to analyze the data obtained by qRT-PCR and expressed as the relative expression of miRNAs by 2^−ΔΔCT^. The Student’s test or the chi-square test was used to compare variables between prostate cancer patients and healthy controls. The ROC curves and the AUC were used to assess the predictive ability of a single miRNA or a combination of miRNAs to diagnosis prostate cancer. The specificity and sensitivity of miRNA were calculated from the Youden index. In addition, multiple logistic regression analysis was used to evaluate the efficiency and reliability of diagnostic panels for prostate cancer screening or diagnosis. A *p*-value of <0.05 implies statistical significance.

## Result

### Screening and testing of candidate miRNAs

**FIGURE 2:**
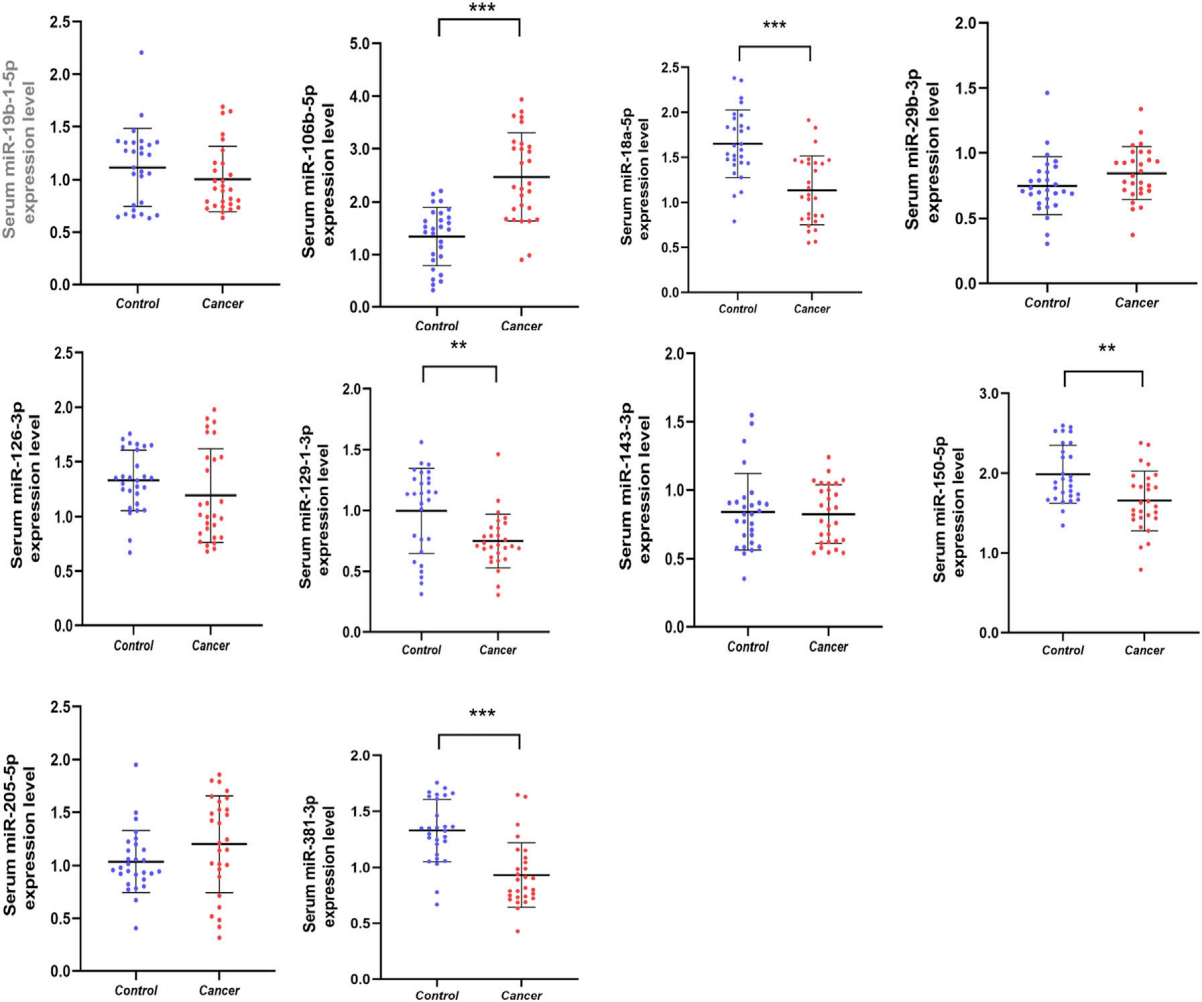
Relative serum expression levels of 10 candidate miRNAs. There was a total of 28 PCas serum samples and 28 NC serum samples utilized in this phrase and 5 miRNAs ultimately showed significant difference. *p < 0.05, **p < 0.01, ***p < 0.001.

At the screening stage, we initially screened 10 miRNAs associated with prostate cancer by finding articles published in the Pubmed database, namely, miR-19b-1-5p, miR-106b-5p, miR-18a-5p, miR-29b-3p, miR-126-3p, miR-129-1-3p, miR-143- 3p, miR-150-5p, miR-250-5p, miR-381-3p (Figure 2). Serum from 28 PCa patients and 28HCs, corresponding to gender and age, were subjected to qRT-PCR to detect the expression levels of these 10 miRNAs in serum. *p*-value <0.05 was used as a screening criterion. Compared with the control group, there were four miRNAs (miR-18a-5p, miR-129-1-3p, miR-150-5p, miR-381-3p) with decreased expression and miR-106b-5p overexpression in PCa patients, and these five miRNAs would be the candidate miRNAs to enter into the subsequent validation stage. The five miRNAs that were not mentioned did not show significant differences and were therefore excluded.

### Validation stage and analysis of diagnostic performance

**FIGURE 3:**
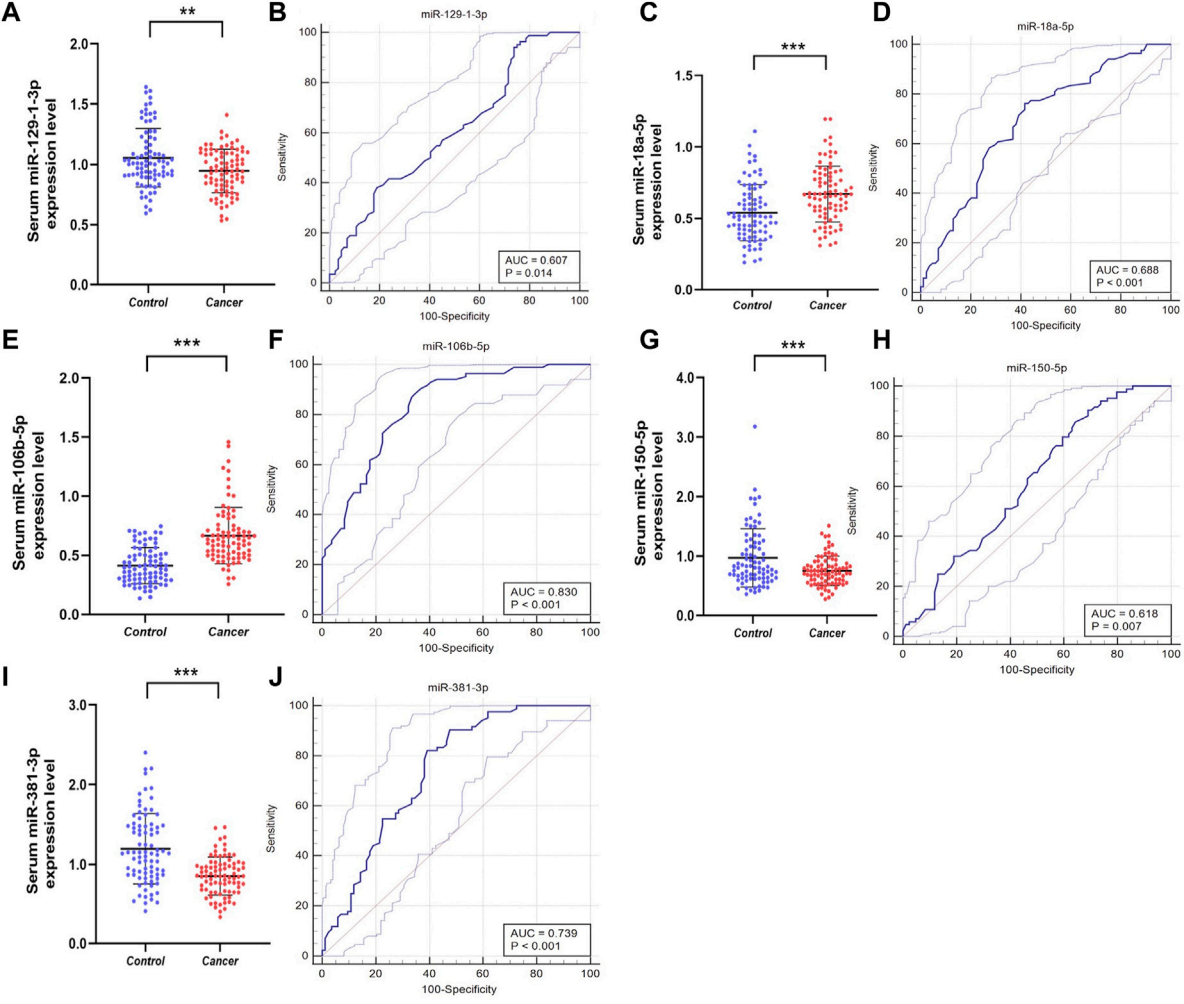
Relative expression counting and receiver operating characteristic curve (ROC) analyses in the validation phase of five elected miRNAs. This phase consisted of 84 PCas and 84 NC serum samples. Area under the curve (AUC) of the five miRNAs were: 0.607 (95% CI: 0.529–0.681, *p *= 0.014 **(B)**) for miR-129-1-3p **(A)**, 0.739 (95% CI: 0.665–0.803, *p *< 0.001 **(J)**) for miR-381-3p **(I)**, 0.830 (95% CI:0.765–0.884, *p *< 0.001 **(F)**) for miR-106-5p **(E)**, 0.688 (95% CI: 0.612–0.757, *p*<0.001 **(D)**) for miR-18a-5p **(C)**, and 0.618 (95% CI: 0.540–0.691, *p *= 0.007 **(H)**) for miR-150-5p **(G)**, respectively. *p < 0.05, **p < 0.01, ***p < 0.001.

To further confirm whether the above candidate miRNAs have diagnostic value, in the validation phase, we expanded the sample size and analyzed serum from 84 PCa patients and 84 HCs. As shown in Figure 3, the results demonstrated that miR-129-1-3p, miR-381-3p expression was decreased, miR-106b-5p was overexpressed, while miR-18a-5p, miR-150-5p expression was not different so they were excluded. Meanwhile, to evaluate the diagnostic ability of individual miRNAs or miRNA combinations in PCa, risk score analysis was used, and receiver operating characteristic (ROC) curves were plotted by sensitivity, false positive rate. Area under the curve (AUC) of the five miRNAs were 0.607 (95% confidence interval (CI): 0.529–0.681) for miR-129-1-3p, 0.739 (95% CI: 0.665–0.803) for miR-381-3p, 0.830 (95% CI: 0.765–0.884) for miR-106-5p, 0.688 (95% CI: 0.612–0.757) for miR-18a-5p, and 0.618 (95% CI: 0. 540 to 0.691) for miR-150-5p, respectively. The cutoff values were derived from the Youden index calculated from the relevant data. Table 2 lists the optimal specificities and sensitivities of these five miRNAs.

**TABLE 2:** Outcomes of receiver operating characteristic curves and Youden index for 5 candidate miRNAs and the three-miRNA panel.

	AUC	*p* value	95% Cl	Associated	Sensitivity	Specificity
				Criterion	(%)	(%)
miR-381-3p	0.739	< 0.001	0.665–0.803	≤1.12	90.48	52.38
miR-106b-5p	0.830	< 0.001	0.765–0.884	>0.44	90.48	63.10
miR-129-1-3p	0.607	= 0.013	0.529–0.681	≤1.18	94.05	26.19
miR-150-5p	0.618	= 0.006	0.540–0.691	≤1.06	90.48	30.95
miR-18a-5p	0.688	< 0.001	0.612–0.757	>0.54	76.19	58.33
Three-miRNA panel	0.912	< 0.001	0.858–0.950	>0.371	91.67	79.76

AUC, area under curve; Cl, confidence interval.

### Construct a diagnostic panel and method

**FIGURE 4:**
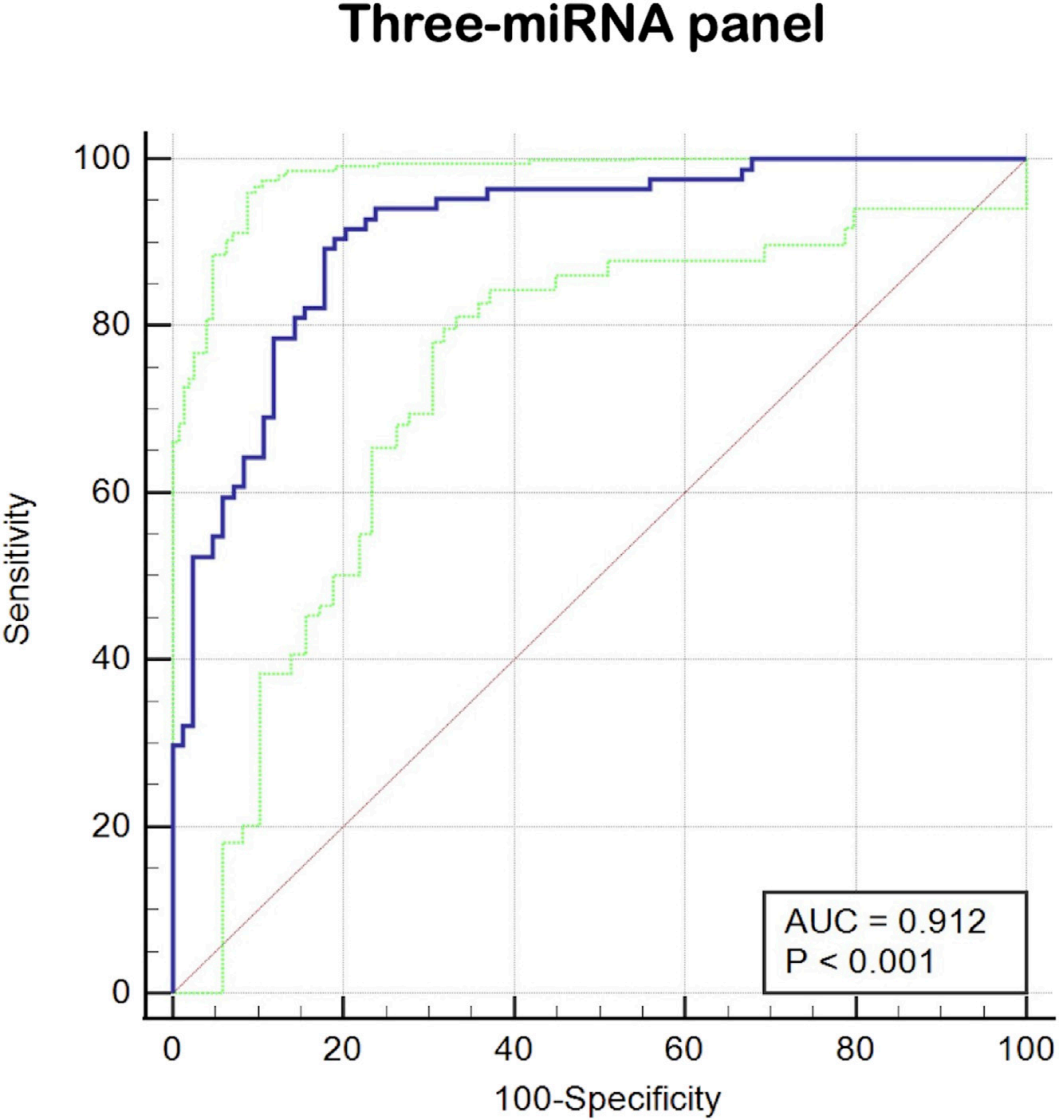
The ROC curve evaluation for the three-miRNA panel. This three- miRNA panel contained miR-129-1-3p, miR-381-3p and miR-106b-5p, and the AUC of three-miRNA panel was 0.912 (95%CI: 0.858 to 0.950; *p* < 0.001; sensitivity = 91.67%; specificity =79.76%).

Since it is more difficult to achieve satisfactory sensitivity and specificity for a single marker, this study combined multiple high-quality miRNAs to improve the diagnostic rate of prostate cancer by building a logistic regression model and analyzing it. The optimal diagnostic model included three miRNAs: miR-129-1-3p, miR-381-3p, and miR-106b-5p. The logistic regression equation for constructing this model was Logit (P) = 1.795 + (10.115 × miR-106b-5p) + (−4.599 × miR-129-1-3p) + (−2.507 × miR-381-3p). As shown in Figure 4, the AUC of three-miRNA panel was 0.912 (95% CI: 0.858 to 0.950; *p* < 0.001; sensitivity = 91.67%; specificity = 79.76%; Figure 4).

### Bioinformatic analysis of candidate miRNAs

**FIGURE 5:**
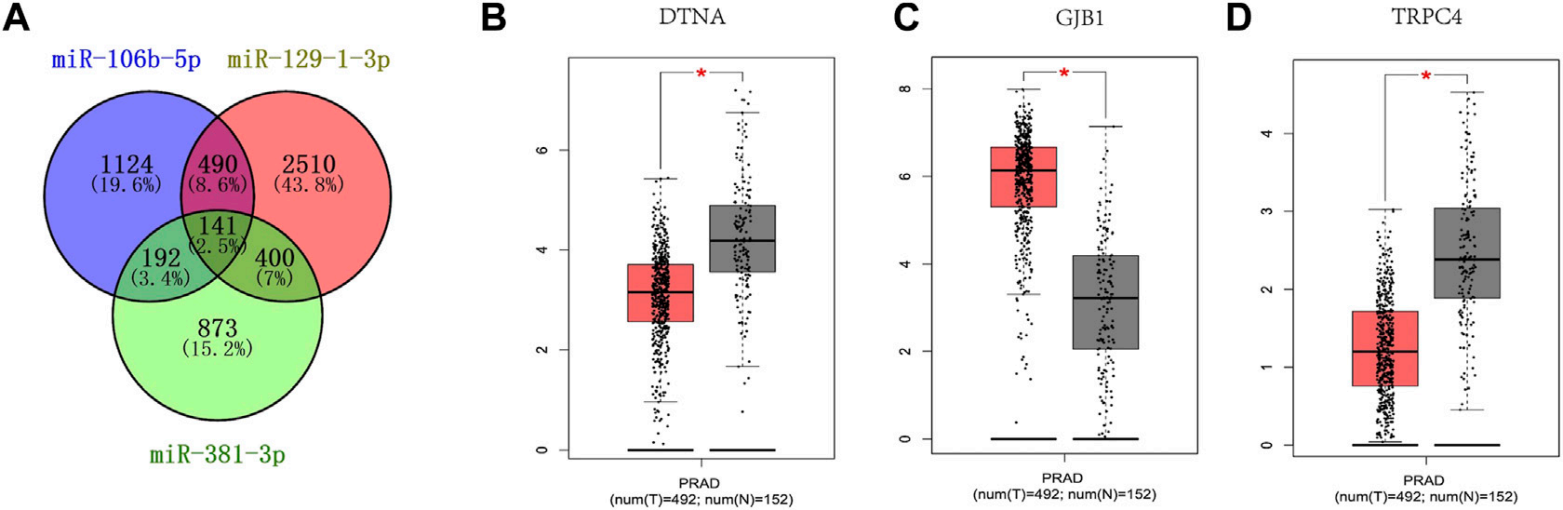
Target genes prediction of the three candicate miRNAs by miRWalk 3.0. Genes that were predicted in over two miRNAs were regarded as potential targets, and eventually, 1223 genes were elected **(A)**. GEPIA was applied to predict these genes relating to three candidate miRNAs in 492 PCas and 192 NCs. DTNA **(B)**, GJB1 **(C)** and TRPC4 **(D)** were dysregulated with |log2FC| > 1 and *p* < 0.01. DTNA **(B)**, GJB1 **(C)** and TRPC4 **(D)** were associated with the prognosis of PCas. T, tumor; N, normal control.

As shown in Figure 5A, the target genes of each of the three candidate miRNAs were predicted by miRWalk 3.0. Based on the screening criteria, a total of 1,223 target genes that could predict two or more miRNAs were selected, of which a total of 141 genes that could co-predict the three candidate miRNAs were selected. The data from the GEPIA database and the TCGA and GTEx programs were analyzed for 141 genes under the criteria of *p* < 0.01 and | log2FC| Cutoff > 1.5 criteria; the analysis of the 141 genes suggested that the expression of DTNA, GJB1 and TRPC4 were significantly different in prostate cancer patients and controls, with decreased expression of DTNA and TRPC4 (Figures 5B, D), and the opposite for GJB1 (Figure 5C). In other words, DTNA, GJB1 and TRPC4 genes can be considered as potential target genes for three-miRNA panel.

**FIGURE 6:**
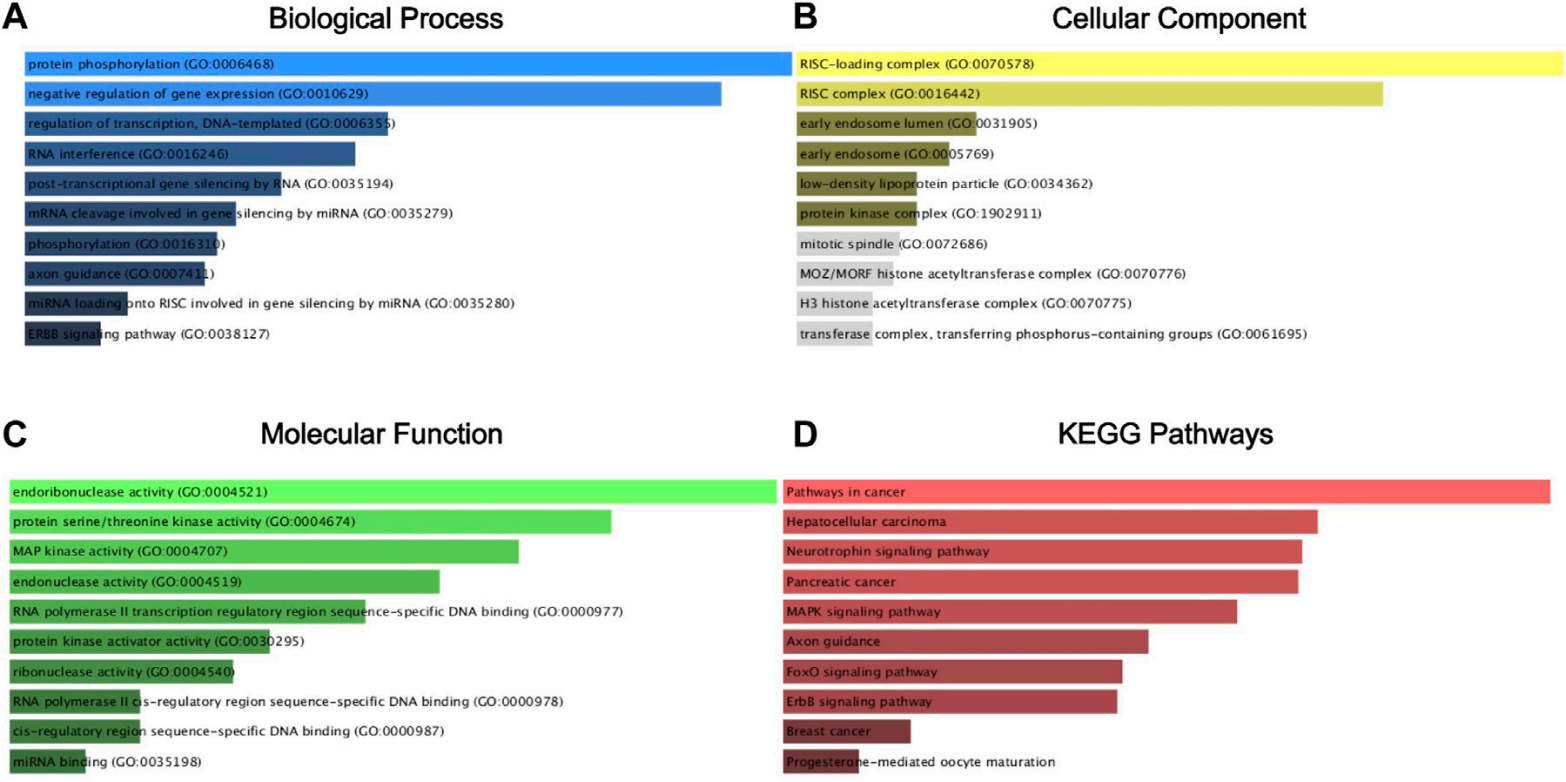
GO annotation and KEGG pathway enrichment agalysis of the target gene by the Enrichr database. Biological process (BP) analysis **(A)**, cellular component (CC) analysis **(B)**, molecular function (MF) analysis **(C)**, and KEGG pathway enrichment analysis **(D)**.

We analyzed the predicted 1,223 target genes for GO annotation and KEGG pathway enrichment by the Enrichr database. As shown in Figure 6, the three GO functional categories, Biological Process, Cellular Component and Molecular Function, were most significantly enriched for protein phosphorylation, RISC-loading complex. Based on the results of KEGG pathway analysis, we also know that the target genes are associated with pathways in cancer, Hepatocellular carcinoma, Neurotrophin signaling pathway, Pancreatic cancer, MARK signaling pathway.

## Discussion

Prostate cancer is a malignant tumor that significantly affects male survival. The pathogenesis of prostate cancer is currently poorly understood and is mainly related to genetics, age, environment, and diet. The early clinical symptoms of prostate cancer are often not obvious and lack specific symptoms, mainly manifesting as lower urinary tract symptoms, which are difficult to distinguish from benign prostatic hyperplasia (BPH). Therefore, early diagnosis of prostate cancer is challenging. The detection of prostate-specific antigen (PSA) in serum, which was first proposed in the 1970s, is of great importance in the screening of prostate cancer (Albertsen, 2020; Carlsson and Vickers, 2020). However, studies have revealed that between 22% and 42% of prostate cancer screening cases in the United States are overdiagnosed, leading to unnecessary treatment (Telesca et al., 2008; Draisma et al., 2009; Loeb et al., 2014). These cases do not require immediate treatment measures, as they may not even experience significant symptoms or benefit from treatment (Vickers et al., 2014). Numerous studies have highlighted the significance of miRNAs in tumorigenesis and progression in various organs of the human body (Li et al., 2013; Kovaříková et al., 2016; Pratap et al., 2018; Swearson et al., 2022). Previous studies have also demonstrated the stability of miRNAs in biofluids such as serum (Enelund et al., 2017). Therefore, miRNA is a high-quality, non-invasive, and low-cost diagnostic biomarker for cancer.

At the beginning of our study, we searched for 10 miRNAs that are aberrantly expressed in prostate cancer through the literature database. We initially considered them to be of possible diagnostic value for prostate cancer. These 10 miRNAs were examined for their diagnostic value in prostate cancer by analyzing the serum of 28 healthy controls and 28 patients with prostate cancer. The five miRNAs (miR-18a-5p, miR-129-1-3p, miR-150-5p, miR-381-3p, miR-106b-5p) were found to be differentially expressed in prostate cancer serum. The sample size was then expanded to 168 enrollees (84 PCa and 84 NC) for further validation. Eventually, three miRNAs (miR-129-1-3p, miR-381-3p, miR-106b-5p) stood out and demonstrated significant diagnostic capabilities. Based on these three high-quality miRNAs, an optimal diagnostic panel was successfully established, which exhibited high sensitivity and strong specificity. Therefore, the combination of these three miRNAs into a panel can be considered as a new biomarker for diagnosing prostate cancer.

After step-by-step validation experiments, it was found that miR-129-1-3p expression was decreased in prostate cancer, suggesting its potential role in inhibiting the development of prostate cancer. MiR-129-1-3p belongs to the miR-129 family, which is involved in the occurrence and development of multiple tumors. Previous studies have shown that miR-129-1-3p may inhibit the migration of the human gastric cancer cell line BGC-823 by targeting and regulating bradykinin receptor B2 (BDKRB2), thereby inhibiting the metastasis of gastric cancer (Wang et al., 2014). MiR-129-1-3p has also been reported to play a role in triple-negative breast cancer by targeting GRIN2D and inhibiting tumor cell growth (Li et al., 2022). However, the pathogenesis of miR-129-1-3p in prostate cancer has not been studied so far. Therefore, this study is the first to demonstrate that miR-129-1-3p can play a role in prostate cancer diagnosis. Further, we will continue investigating the possible mechanisms of miR-129-1-3p in regulating prostate cancer development.

In our current study, we could observe decreased expression of miR-381-3p in prostate cancer cells. Perhaps, we can regard it as an oncogene to regulate the growth of prostate cancer cells negatively. In 2019, a study illustrated a significant decrease in the expression of UBE2C, which acts to regulate tumor cell proliferation and increase tumor invasiveness, after high expression of miR-381-3p was observed in LNCaP and PC-3 cells (Williamson et al., 2009; Hu et al., 2019). In other words, miR-381-3p impeded the expression and function of UBE2C to curb the progression of prostate cancer. In bladder cancer, miR-381-3p has also been found to regulate the cell cycle of bladder cancer cells, increasing apoptosis rate by targeting negative regulation of CDK6, CCNA2, and MET (Li et al., 2019). Similarly, previous studies in cervical cancer (Shang et al., 2019), colorectal cancer (Wang et al., 2022), and thyroid cancer (Kong et al., 2018) have found that miR-381-3p inhibits the metastasis and proliferation of tumor cells. In combination with these studies, perhaps we can use the expression of miR-381-3p in tissues and cells as an indicator for diagnosis and monitoring tumor recurrence and prognosis.

In the diagnostic panel we constructed, miR-106b-5p was the only miRNA overexpressed in prostate cancer cells. Previous studies have reported the association between miR-106b-5p and prostate cancer. An experiment conducted in Indonesia demonstrated that local prostate cancer patients have higher expression levels of miR-106b-5p compared to BPH patients and healthy individuals. It was found that miR-106b-5p is involved in interfering with the endoplasmic reticulum stress repair pathway and repressing oncogenes to regulate prostate cancer (Bonnu et al., 2021). In another study, researchers constructed a miRNA-mRNA regulatory network containing miR-106b-5p to identify genes such as TMEM100, FRMD6, NBL1, and STARD4 for the diagnosis and prognosis of prostate cancer patients. They concluded that signaling pathways such as cGMP-PKG, PI3K-Akt, and cAMP are involved in regulating prostate cancer progression (Su et al., 2022). Among the five single miRNAs studied, miR-106b-5p demonstrated the best diagnostic ability, exhibiting excellent sensitivity and specificity (90.48% and 63.10%, respectively). These studies provide evidence for miR-106b-5p as a potential diagnostic biomarker for prostate cancer.

In addition to the successful constructing of diagnostic panels, our study also focused on identifying potential target genes. Three genes, DTNA, GJB1, and TRPC4, were identified as target genes. DTNA encodes a protein component of the dystrophin-associated protein complex, and mutations in this gene are primarily associated with left ventricular dystrophy. Current studies have also shown that DTNA is associated with bladder cancer (Shi et al., 2022), liver cancer (Hu et al., 2020), oesophageal cancer (Fu et al., 2021) and colorectal cancer (Liu et al., 2018). In bladder cancer, overexpressed DTNA functions as an oncogene and, along with four other genes, forms a diagnostic model for detecting recurrence and prognosis (Shi et al., 2022). However, in esophageal cancer, low expression levels of DTNA were negatively correlated with miR-301b and positively correlated with the prognosis of esophageal cancer patients, suggesting an anti-oncogene role (Fu et al., 2021). In our study, the expression of DTNA was also low in prostate cancer tissues, and we will further test whether the expression level of DTNA is related to the prognosis and recurrence of prostate cancer. GJB1 (gap junction protein beta-1) encodes Cx23 (connexin32), which forms gap junctions (GJs) that facilitate the transfer of ions and small molecules between cells. High expression of GJB1 in prostate cancer inhibits prostate cancer invasion and metastasis and is considered the most predictive prostate cancer marker in urine (Yun et al., 2015; Yazbek Hanna et al., 2023). Additionally, the literature shows that Cx23 increases EGFR levels by upregulating Src expression and inhibits apoptosis in hepatocellular carcinoma cells by inducing activation of the EGFR signaling pathway (Xiang et al., 2019). This also provides ideas for our subsequent experiments. Currently, GBJ1 has been less studied in prostate cancer, making it a promising marker. TRPC4 is a member of the TRP family, and TRP channels are ion channels highly permeable to cations, especially Ca2+ (Nilius and Owsianik, 2011). Studies have demonstrated that Ca2+ regulates tumor development by affecting various biological activities such as cell proliferation and apoptosis (Canales et al., 2019). A literature published in 2019 suggests that TRPC4 can be used as an independent influencing factor to determine the risk of recurrence after radical prostate cancer surgery: high expression of TRPC4 is associated with a lower risk of recurrence (Perrouin-Verbe et al., 2019). The role of TRPC4 in tumor biological activities is a current research hotspot, and research in prostate cancer still needs to be further expanded.

## Conclusion

In our study, we looked for three serum miRNAs that could be used for prostate cancer screening and early diagnosis. Moreover, we successfully constructed a diagnostic panel with excellent diagnostic performance, sensitivity and specificity using these three miRNAs, which is expected to move to the next step of clinical research and accelerate the use of tumor markers in the clinic. In addition, based on the above three miRNAs, we also found three target genes (DTNA, GJB1, and TRPC4) with the ability to predict tumors.

## Data Availability

The original contributions presented in the study are included in the article/supplementary material, further inquiries can be directed to the corresponding author/s.
